# 5-Azacytidine and decitabine induce C > G transversions in both murine and human cells

**DOI:** 10.1038/s41375-025-02670-y

**Published:** 2025-07-18

**Authors:** Ryan Bertoli, Dengchao Cao, Olivia Tuckey, Susannah Gammell, Anthony Wokasch, Yang Jo Chung, Jason M. Foulks, Peter D. Aplan

**Affiliations:** 1 Genetics Branch, Center for Cancer Research, National Cancer Institute, National Institutes of Health, Bethesda, MD USA; 2 Myeloid Malignancies Program, National Institutes of Health, Bethesda, MD USA; 3 Sumitomo Pharma Oncology, Inc, Marlborough, MA USA

**Keywords:** Chemotherapy, Cancer genetics, Acute lymphocytic leukaemia, Myelodysplastic syndrome, Oncogenes

## Abstract

5-Azacytidine (5AZA) is a DNA methyltransferase inhibitor (DNMTi) used clinically to treat myelodysplastic neoplasm (MDS), and is used off-label for a number of malignancies including acute myeloid leukemia. This cytidine analog depletes intracellular DNMT1, and it has been hypothesized that DNMT1 depletion leads to hypomethylation and de-repression of methylated tumor suppressor genes. We used a pre-clinical model of MDS to investigate the efficacy of 5-azacytidine. Unexpectedly, we found an increased frequency of acute lymphoid leukemia (ALL) in 5AZA treated mice. Whole exome sequencing (WES) revealed a large number of C > G transversions in 5AZA treated mice, including genes known to be important for ALL such as *Chd4, Ikzf1*, and *Trp53*. Single base substitution (SBS) profiling revealed increased C > G mutations in the ALL cells, with a mutation signature similar to the previously described SBS39 signature. An in vitro GEMINI (Genotoxic Mutational Signature Identified After Clonal Expansion In vitro) assay recapitulated the finding of increased C > G mutations in both murine and human cell lines. Furthermore, similar GEMINI assays revealed induction of C > G mutations in cells treated with decitabine. Taken together, these findings demonstrate that azanucleosides induce C > G mutations both in vitro and in vivo, and are linked to leukemic transformation in murine cells.

## Introduction

Myelodysplastic neoplasm (MDS) represents a heterogeneous group of blood cancers characterized histologically by dysplastic cell morphology and clinically by pancytopenia [1, 2]. MDS can be further stratified into risk groups, which predict clinical course, outcome, and risk of transformation to acute myeloid leukemia (AML), an aggressive and often fatal blood cancer [3]. In the highest risk MDS group, the risk of transformation to AML is ~50%, and this transformation carries a 2-year survival of around 9% [4–6]. Methylation of cytidine residues is a key regulator of normal gene expression and differentiation, however, hypermethylation of cytidine residues is associated with both MDS and AML [7, 8]. Specifically, it has been hypothesized that inappropriate methylation of the promoter region of tumor suppressor genes can drive malignant transformation [7].

DNA MethylTransferase Inhibitors (DNMTi), a class of drugs that inhibit DNA methylation, are often employed clinically to reverse global hypermethylation [9]. These drugs are thought to function by binding irreversibly to DNMT1, leading to enzyme degradation by proteases [7]. The depletion of DNMT1 leads to global cellular hypomethylation, which is hypothesized to reactivate tumor suppressor genes that have been silenced via hypermethylation, thereby leading to tumor inhibition and apoptosis [10]. The two DNMTi approved for use in MDS by the US Food and Drug Administration (FDA) are 5-azacytidine (5AZA) and the deoxy derivative of 5-azacytidine [5-aza-2′-deoxycytidine or decitabine (DAC)] [10]; 5AZA can be regarded as a prodrug for DAC, which is capable of incorporation into DNA. Additionally, these two drugs have been used off-label for other hematological malignancies such as AML [11, 12]. In this study, we sought to determine the efficacy of 5AZA, with or without alvocidib (ALVO), using a murine model of MDS driven by a *NUP98::HOXD13* (*NHD13*) fusion gene [13].

## Materials and methods

### Ethic approvals and consent to participate

No human subjects were involved in this study. All animal experiments were were performed in accordance with the relevant guidelines and regulations and approved by the National Cancer Institute (Bethesda, Maryland, U.S.A.) Intramural Animal Care and Use Committee (ACUC), protocol numbers MB-057, GB-006, and GB-007.

### Mouse strains, genotyping, and identification

Transgenic mice expressing the *NUP98::HOXD13* (*NHD13*) transgene were generated on a C57BL/6 background and genotyped as previously reported [13]. These mice expressed the *Cd45* allele *Cd45.2*; whereas the recipient congenic C57BL/6 mice expressed the *Cd45.1* allele. Transplant recipients were both male and female and were purchased from Jackson Laboratory. The sample size was powered to detect a 40 percent difference in survival; any mice that died following transplant but before beginning drug treatment were excluded. Mice were treated based on cage assignment; there was no other randomization. Animal caretakers who reported sick or moribund mice were blinded as to treatment groups; the investigators were not blinded.

### Drug treatment and schedule

5-azacytidine (5AZA, CAS: 320-67-2) was obtained from Sigma-Aldrich (CAT# A2385) in a powdered form, and stock solutions of 2.05 uM were prepared and stored as single-use aliquots at −20 °C. 5-aza-2′-deoxycytidine (Decitabine or DAC, CAS: 2353-33-5) in powdered form was obtained from Sigma-Aldrich (Cat#1165204), and stock solutions of 0.468 uM were prepared and stored as single-use aliquots at −20 °C. Alvocidib (ALVO, Flavopiridol, CAS: 131740-09-5) was obtained as a hydrochloride hydrate in powder form from Sigma-Aldrich (Cat# F3055) and stock solutions of 1.9 uM were prepared and stored as single-use aliquots at −20 °C. Once thawed, aliquots of any drug would be used within an hour and any remaining drug was discarded. In vivo experiments utilized a 4-week cycle of treatment. After disinfection of the anterior abdominal wall with ethanol, mice were injected intraperitoneally with 5AZA (2 mg/kg), or sterile phosphate buffered saline (PBS) control. During injection, mice were placed in a supine position as to minimize the risk of abdominal organ puncture. In the first week of treatment, 5AZA or PBS injections would be repeated for a total of five days, followed by 2 days of rest. In the 2^nd^ week, a single injection of either ALVO (5 mg/kg) or PBS was given in the first day, followed by 20 days of rest. This treatment schedule constituted one treatment cycle, which would be repeated until mice succumbed to disease, or as many as 16 cycles.

### Leukemia assessment

Complete blood counts (CBCs) were collected via tail vein prior to beginning each treatment cycle and assayed on a HEMAVET Multispecies Hematology Analyzer (CDC Technologies). Mice were assessed for signs of illness such as lethargy, cachexia, hunched posture, ruffled fur, and non-responsiveness. Mice with clear signs of illness were euthanized via CO_2_ asphyxiation. Mice that were euthanized or found dead were assessed for the presence of organomegaly, tumor mass, or effusion. Spleen, thymus, liver, and bone marrow were collected and suspended in Hank’s balanced salt solution supplemented with 2% fetal bovine serum (HF2). Bone marrow from femur and tibia were flushed with HF2, and portions of parenchymal organs were placed in 10% formalin. Hematological malignancies were diagnosed according to published guidelines [14].

### Flow cytometry analysis

Flow cytometry was performed as described previously [15]. Single cell suspensions of spleen and thymus were generated by gently disaggregating the tissue using a pestle and then filtering the cells through a 40-uM mesh filter in HF2. Single cell solutions were resuspended in HF2 with 5% rat serum (used to block non-specific binding by Fc receptors). A cocktail of antibodies was added to each single cell suspension and incubated for at least 30 min at 4 degrees Celsius. The antibody cocktail included: Mac-1/PE (clone-M1/70), BioLegend; Cd71/PE/Dazzle594 (clone-R172170), BioLegend; Cd45.2/Alexa Fluor 532 (clone-104), Invitrogen; B220/PerCP-eFluor 710 (clone-RA3-6B2), Invitrogen; Sca-1/PE-Cy7 (clone-D7), eBioscience; Ter119/APC (clone-TER119), BioLegend; Cd19/Alexa Fluor 700 (clone-6D5), BioLegend; 7AAD (Viability), BioLegend; Kit/APC-eFluor 780 (clone-2B8), Thermo Fisher; Cd4/PerCP-Cy5.5 (clone-GK1.5), BioLegend; and Cd8/APC-Cy5.5 (clone-53-6.7), Abcam. Cells were washed in PBS after incubation and resuspended in HF2 for analysis. Cytometry was conducted using a Cytek Northern Lights cytometer (Supplementary Fig. S1).

### Histology and immunohistochemistry (IHC)

Organs for immunohistochemistry (IHC) were placed in 10% formalin and then embedded in paraffin. Paraffin sections were stained with hematoxylin and eosin (H&E), myeloperoxidase (Cat#A0398; Dako), and Cd3 (Clone-Cd3-12, Bio-Rad). Sections were scanned using an Aperio At2 digital slider scanner (Leica Biosystems) and stored in a database within the eSlide Image Management System. Scans were viewed using Aperio ImageScope (RRID:SCR_020993) v12.4.6.5003 software.

### Cell culture

The human U937 cell line was obtained from American Type Culture Collection (ATCC). 7298 is a murine T-ALL cell line derived from *Scl/Lmo1* transgenic mice as previously described [16]. 961C is a murine myeloid leukemia cell line generated from *NUP98::PHF23* (*NP23*) transgenic mice [17]. T-259 is a murine B-cell precursor acute lymphoblastic leukemia cell line generated from *NP23* transgenic mice [18]. 748T is a T-cell precursor acute lymphoblastic leukemia cell line generated from *NP23* transgenic mice [17]. Cell lines were authenticated by presence of known fusion genes (CALM::AF10 for U937; NUP98::PHF23 for murine cell lines). Cell lines were not tested for mycoplasma contamination. All cell lines were cultured as single cell suspension in Iscoves modified Dulbecco Media (IMDM) media supplemented with 10% fetal bovine serum (FBS), 1% glutamine, and 1% penicillin/streptomycin. Cell lines were maintained as previously reported [16, 18].

### In vitro drug treatment (GEMINI assay)

GEMINI was conducted as previously described [19]. Cell counts and cell viability were assessed using the TC20 Automated Cell Counter (BioRad) with Trypan Blue as a reporter dye (Lonza BioWhittaker Trypan Blue 0.4%, Fisher Scientific, Cat# BW17-942E).

### Nucleic acid extraction, polymerase chain reaction, and Sanger sequencing

DNA was extracted using the DNeasy Blood & Tissue kit (Qiagen) and the manufacturer’s recommended protocol. Polymerase Chain Reaction (PCR) was utilized to characterize clonal murine T cell receptor beta rearrangements using DNA primers and protocols as previously described [20]. DNA quality control was assessed by PCR amplification of the murine *Scid* locus. PCR assays utilized the LiTaq PCR mix (Cat# M0024, LifeSct) and the manufacturer’s recommended protocol. Sanger sequencing of purified DNA amplification products was performed by the NCI Sequencing Core. *Trb* rearrangements characterized by PCR were confirmed by inspection of WES. bam files using an Integrated Genome Viewer (IGV, Broad Institute v 2.17.4).

### Whole-Exome Sequencing (WES) and mutation signature analysis

Genomic DNA concentration was measured by Qubit 1x dsDNA-HS assay kit (Invitrogen, cat # Q33231). 200 ng of DNA were sheared to ~175 bp using a Covaris Instrument (E220 PLUS) with the following parameters: duty factor 25%; Peak Incident power 220; cycle burst 50; time-200 s at 4 °C. Agilent SureSelectXT library prep ILM Kit (Agilent # G9611B) was used to prepare the library for each sheared mouse DNA sample. DNA fragments’ ends were repaired, followed by Adenylation of the 3’ end and then ligation of paired-end adapter. Adapter-Ligated libraries were then amplified (Pre-capture PCR amplification: 98 °C 2 min, 10 cycles: 98 °C 30 s, 65 °C 30 s, 72 °C 1 min, then 72 °C 10 min, 4 °C hold) by Herculase II fusion enzyme (Agilent Technologies, # 600679). After each step DNA was purified by Ampure XP beads (Beckmann Coulter Genomics # A63882). Samples were analyzed on an Agilent Tapestation 4200, DNA-1000 kit (Agilent # 5067-5582). The concentration of each library was determined by integrating under the peak area of ~200–500 bp. Each gDNA library (~750–1000 ng) was hybridized with biotinylated mouse specific capture RNA baits (SureSelectXT Mouse All Exon, catalog # 5190-4642, 96 reactions) in the presence of blocking Oligonucleotides. Bait-target hybrids were captured by streptavidin-coated magnetic beads (Dynabeads MyOne Streptavidin T1, Life Technologies, # 6560) for 30 min at room temperature. The captured library was eluted in nuclease free water and half volume was amplified with individual index (Post-capture PCR amplification: 98 °C 2 min, 10 cycles: 98 °C 30 s, 57 °C 30 s, 72 °C 1 min, then 72 °C 10 min, 4 °C hold). The libraries were quantitated by qPCR using the Library Quantification Kit v2 – Illumina Genome Analyzer – Universal kit (KK4824 07960140001) from KAPA BIOSYSTEMS INC, and equimolar concentration of the libraries was pooled before sequencing on Illumina’s NovaSeq 6000 sequencer using the S2 flowcell and paired end (2 × 150) cycle sequencing.

The sequencing run was demultiplexed using Illumina Bcl2fastq v2.20 software tool. Raw read sequences were trimmed of adapters and mapped against mouse reference genome mm10 (GRCm38) using Illumina DRAGEN pipeline (version 3.7.5) with target capture BED file (S0276129_Regions_mm10.bed from the Agilent SureSelect XT Mouse All Exon Kit) to specify the exome target regions. The variant calling was performed using DRAGEN in somatic variant calling with pair mode (tumor vs. normal) using the DRAGEN pipeline default parameters. Both small variant analysis (SNV) and large structural variant (SV) analysis were performed using DRAGEN pipeline. Further filtering for quality control (FILTER = ”PASS”) and minimal number of variant reads (AD_TUMOR > = 3). The filtered variants were used for the Mutational Signature Analysis. Mutational matrices for SBS were generated using SigProfilerMatrixGenerator (https://github.com/AlexandrovLab/SigProfilerMatrixGenerator) with default options (version 1.2). Mutational signatures were extracted and decomposed into COSMIC reference signatures using SigProfilerExtractor (https://github.com/AlexandrovLab/SigProfilerExtractor) with default options (v1.1.24) [21].

### Image generation

Chemical structure images were generated using MolView v2.4 (https://molview.org/).

### Statistics and hypothesis testing

Data are reported as means ± standard deviation unless otherwise noted. Graphs were generated using GraphPad Prism v10.2.3 (RRID:SCR_002798) (GraphPad Software, LLC). Survival curves were analyzed with the Mantel–Cox Log-Rank test for significance. Comparison between mutation counts were analyzed using a two-sided Student’s *T* test, except where noted. In this study, *P*-values of less than 0.05 were considered significant.

## Results

### 5-Azacytidine (5AZA) treatment does not increase survival in a preclinical model of MDS

**Fig. 1:**
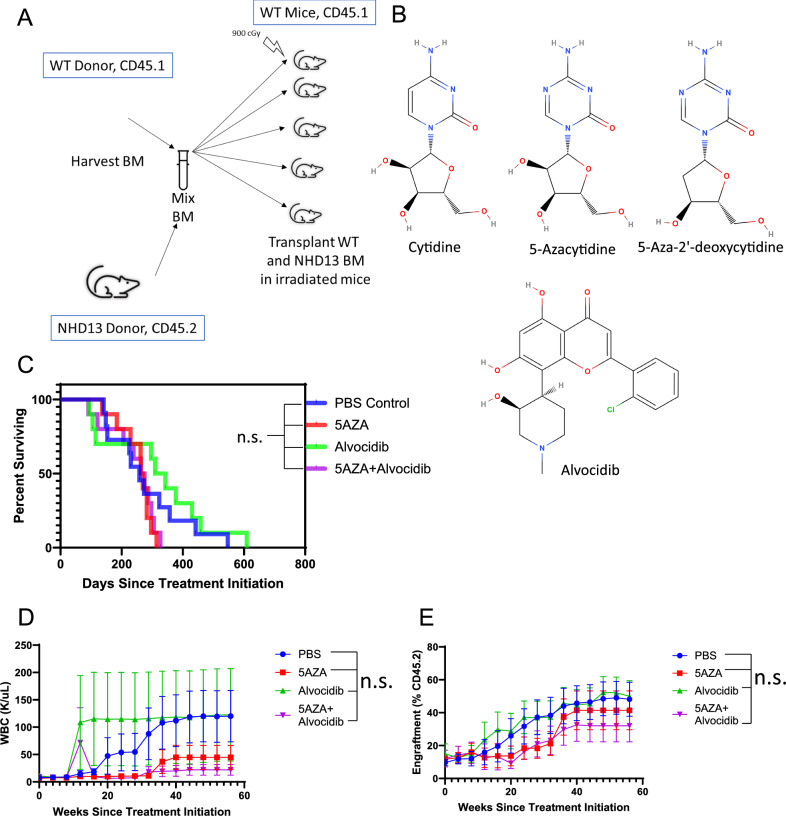
Survival and outcomes of *NHD13* chimeric mice treated with alvocidib and/or 5AZA. **A** Schematic depicting the *NHD13* chimeric transplantation model of MDS/AML. **B** Chemical structures of cytidine, 5-Azacytidine, 5-Aza-2’-deoxycytidine, and alvocidib. **C** Pooled survival and cause of death for the two experiments. **D** WBC count averaged by treatment group over time. **E** *NHD13* engraftment in the peripheral blood averaged by treatment group over time.

*NHD13* transgenic mice typically develop MDS by 6–12 months of age [13]. However, since these mice are transgenic, they have no wild-type (*WT*) cells with normal hematopoiesis. Therefore, we generated chimeric mice with both MDS (*NHD13*) and *WT* hematopoiesis by transplanting 1 ×106 *NHD13* bone marrow nucleated cells (BMNC) and 2 ×105 *WT* BMNC into a cohort of WT mice. The *NHD13* cells express the Cd45.2 isoform of Cd45, while the WT cells express the Cd45.1 isoform, allowing us to distinguish *NHD13* and WT cells via flow cytometry (Fig. 1A). To assess the efficacy of 5-Azacytidine (5AZA) with or without alvocidib (ALVO), a cyclin dependent kinase inhibitor [22], NHD13/WT chimeric mice were treated with 5AZA, ALVO, 5AZA + ALVO, or vehicle (PBS) control (Fig. 1B). There was no difference in survival between the four groups (Fig. 1C), although there was a non-significant decrease in WBC in the 5AZA+/− ALVO treatment groups (Fig. 1D). Peripheral blood engraftment showed a gradual increase in the percentage of *NHD13* cells in all four groups, reflecting the ability of *NHD13* cells to outcompete WT cells (Fig. 1E).

### A subset of 5AZA treated mice develop T-cell acute lymphoblastic leukemia (T-ALL), in *NHD13* or *WT* cells

**Fig. 2:**
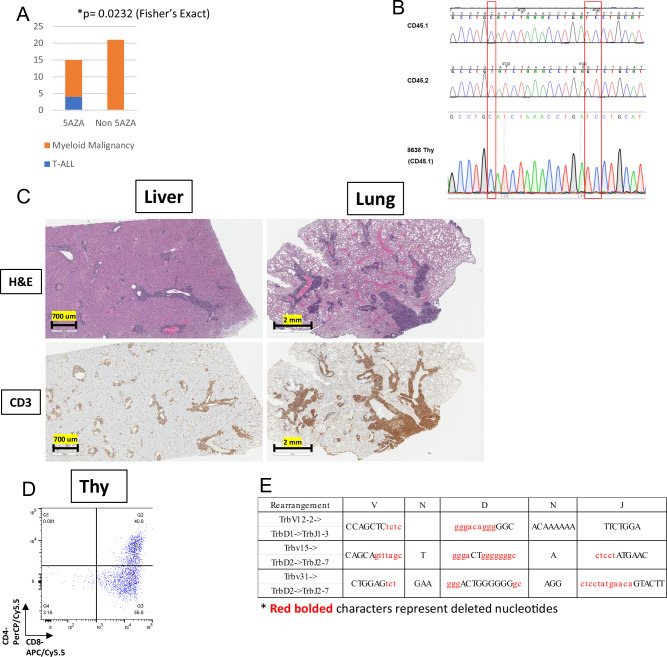
Characteristics of recipient T-ALL treated with 5AZA. **A** Proportion of AML and ALL versus 5AZA exposure. **B** Sanger sequencing demonstrating that malignancy is of WT (Cd45.1) origin. **C** H&E staining of liver and lung. Note peri-portal and perivascular invasion of Cd3 positive lymphoblasts. **D** Flow cytometry demonstrating Cd8^+^CD4^heterogeneous^ cells. **E** T cell receptor beta (*Trb)* rearrangements detected in leukemic cells.

As anticipated, most mice in this study died of a myeloid malignancy (MDS or AML) (Supplementary Table S1A, Supplementary Fig. S2), suggesting that the 5AZA + ALVO combination was not effective in this pre-clinical model. Surprisingly, four mice, all in a 5AZA treated group, died of T-cell acute lymphoblastic leukemia (T-ALL) (Fig. 2A, Supplementary Table S1A). Consistent with T-ALL, all four mice showed an enlarged thymus on necropsy (Supplementary Table S1A). However, although three of the T-ALL were of *NHD13* origin (Supplementary Table S1A, Supplementary Fig. S3), one T-ALL was of WT origin (Supplementary Table S1A, Fig. 2B). Consistent with the diagnosis of T-ALL, this sample showed invasion of liver and lung with T-lymphoblasts (Fig. 2C), expression of Cd4 and Cd8 (Fig. 2D) and clonal Trb gene rearrangements (Supplementary Table S1A, B, Fig. 2E). Similar to the approach used in clinical studies of rare but serious side effects, such as therapy-related leukemia [23–27], we grouped mice into 5AZA exposed (5AZA and 5AZA + ALVO) and 5AZA non-exposed (ALVO and PBS) groups.

### Whole-exome sequencing reveals a marked increase in C > G transversions in T-ALL from 5AZA treated mice

**Fig. 3:**
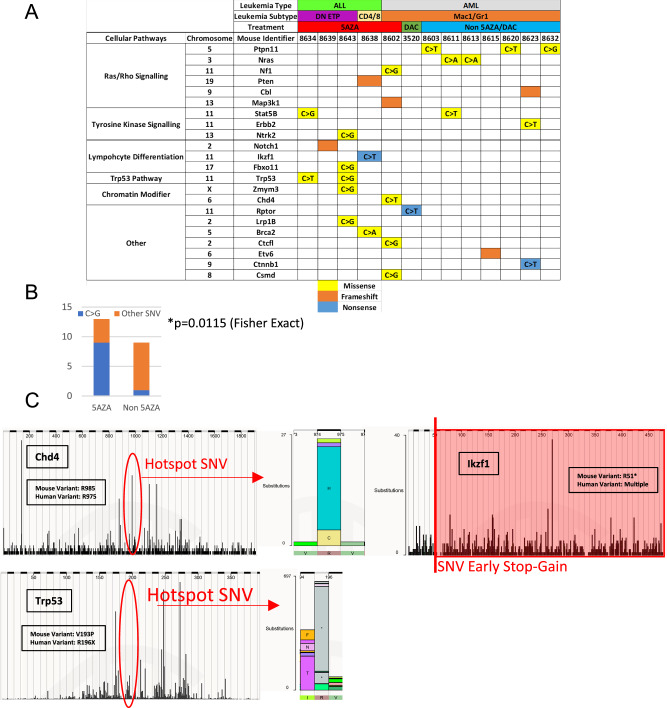
Mutations associated with leukemia in *NHD13* chimeric mice. **A** Mutations organized by leukemic type and subtype, treatment, and lesion type. **B** Increased C > G mutations in leukemia-relevant genes following 5AZA treatment, from data shown in (**A**). **C** Mutations in 5AZA associated leukemias match lesions known in human leukemia, including Chd4 R985X; Ikzf1 R51*; and Trp53 R196X.

We used whole-exome sequencing (WES) to identify acquired mutations that may have led to leukemic transformation (Supplementary Table S2A–C). Candidate driver mutations were identified by comparison with the FoundationOne® Heme Gene panel (Foundation Medicine, Inc.), which consists of 431 genes known to be recurrently mutated in blood cancers (Supplementary Table S3). Candidate driver mutations and related cellular pathways are summarized in Fig. 3A. Interestingly, we noticed an increased number of C > G transversions in mice receiving 5AZA (Fig. 3A). This frequency of C > G SNV was significantly different versus non 5AZA leukemias (Fisher Exact test *p* = 0.0115; Fig. 3B). Additionally, mutations in 5AZA treated leukemias were found at specific residues known to be recurrently mutated in human cancer, such as *Chd4* R975; *Ikzf1* R51*; and *Trp53* R196 (Fig. 3C), supporting the suggestion that these are driver mutations.

**Fig. 4:**
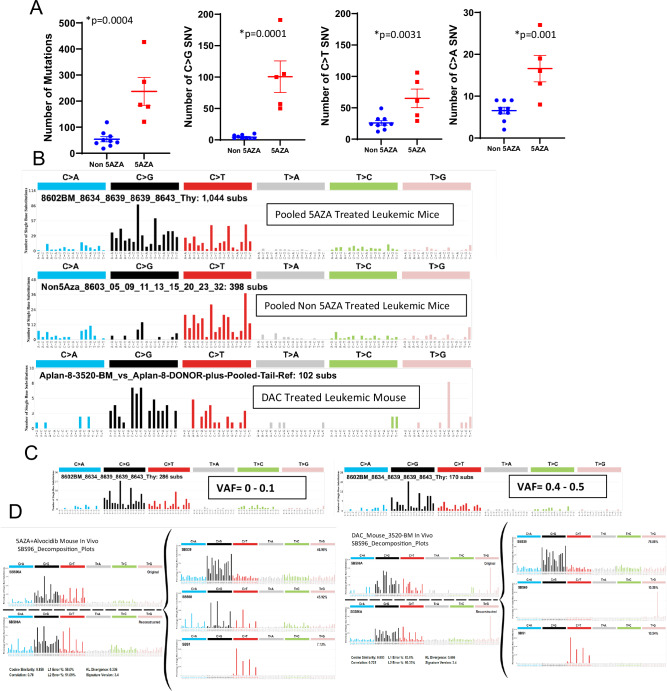
Leukemic cells from 5AZA treated mice reveal a unique SBS mutational profile. **A** Scatterplots demonstrate an increase in total mutations, C > G and C > T SNV’s in 5AZA exposed mice versus non-exposed mice. **B** SBS profile of pooled 5AZA leukemic samples revealing a unique mutational signature with a preference for C > G and C > T mutations vs. non 5AZA control leukemic profiles. **C** SBS profiles partitioned by VAF reveal no notable difference in mutational profile attributable to VAF. **D** SBS decomposition plots demonstrating similarity of the 5AZA and DAC SBS signatures to known SBS signatures.

Based on these results, we asked whether there was an increased frequency of C > G transversions throughout the exome of 5AZA treated mice. We found a clearly increased number of total mutations, including both SNV’s and indels, in 5AZA treated mice versus non exposed mice (*p* = 0.0004)(Fig. 4A). Strikingly, the most common form of mutation was a C > G transversion, with a mean of 101 C > G mutations in 5AZA treated leukemias compared to only four C > G mutations in non-5AZA exposed tumors (*p* = 0.0001). In addition, C > T transitions (*p* = 0.0031) and C > A transversions (*p* = 0.001) were increased as well, but to a lesser extent (Fig. 4A).

Given the increased number of C > G transversions, as well as the smaller number of C > T transitions and C > A transversions, we used SigProfiler software to examine the mutational profile of these leukemias. A Single Base substitution (SBS) analysis of leukemia from the four mice treated with 5AZA shows a signature with a preference for C > G transversions, most commonly in a 5’-NCG-3’ context, compared to leukemia from the non-5AZA leukemia samples (pooled data is shown in Fig. 4B, and individual leukemia data is shown in Supplementary Fig. S4). Additionally, given the structural similarity of 5AZA to DAC (Fig. 1B), we analyzed the mutational profile of a single myeloid leukemia in a mouse treated with DAC in a separate experiment using *NHD13*/*WT* chimeric mice. This profile is similar to that of the 5AZA signature. Given that we used an AD_TUMOR of ≥3, but no variant allele frequency (VAF) cutoff to identify mutations in this study (see methods), it is possible that low VAF SNV may have been sequencing artefacts. However, as shown in Fig. 4C, the mutational signature at low VAF was indistinguishable from the signature noted at high VAF.

We further analyzed the signature of the 5AZA mice and DAC mouse using mutational signature decomposition. This type of analysis attempts to “decompose” an observed mutational profile into various proportions of known SBS signatures catalogued in the Wellcome Sanger Institute’s Catalogue of Somatic Mutations in Cancer (COSMIC) SBS database [28]. Known SBS contribution proportions are determined by how well the original plot is “reconstructed” using only known SBS signatures. The decomposition plot of the four pooled 5AZA leukemic samples reveals major contributions of SBS39 and SBS98, whereas the decomposition plot of the single DAC leukemic sample reveals a major contribution of SBS39 (Fig. 4D). Of note, the SBS98 signature is similar to the SBS-ATC signature that we recently reported in T-cell and B-cell ALL which develops after in vivo treatment of mice with 5-aza-4’-thio-2’-deoxycytidine (ATC), a close analog of 5AZA and DAC [19].

### The GEMINI assay reveals increased mutations and C > G transversions in murine cells treated with 5AZA or DAC in vitro

**Fig. 5:**
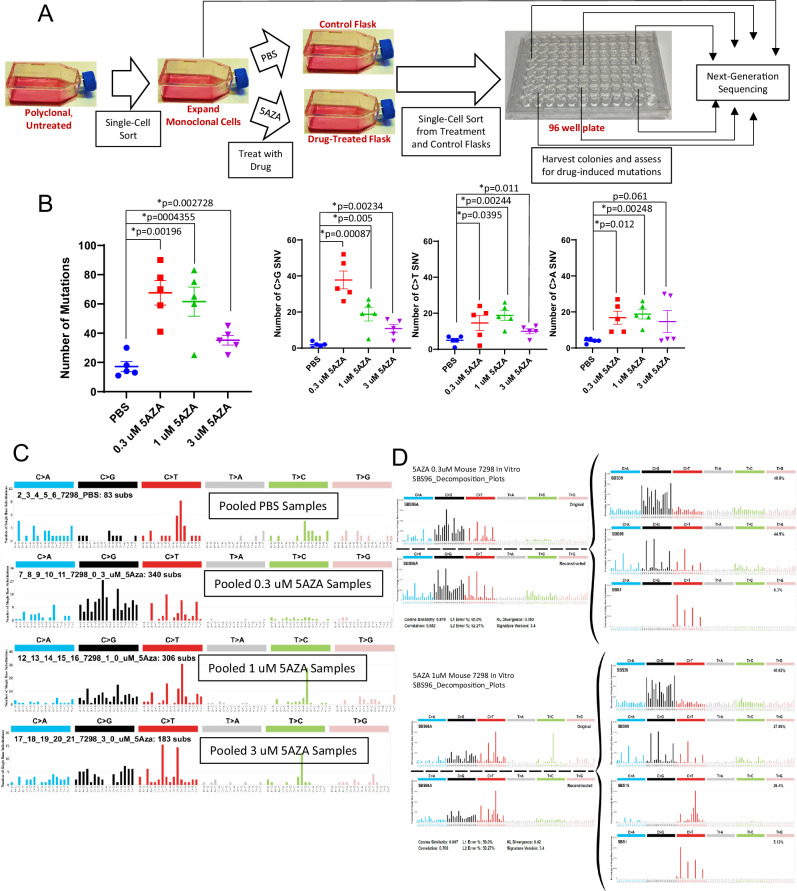
5AZA treatment leads to increased C > G transversions in vitro. **A** Schematic of the GEMINI assay. **B** WES reveals increase in total, C > G, and C > T mutations in 5AZA treated 7298 cells, in an inverse dose-response fashion. **C** SBS plots reveal C > G and C > T signature similar to in vivo results in 5AZA treated samples. **D** SBS decomposition plots demonstrating similarity of the 5AZA SBS signatures to known SBS signatures.

To independently confirm that 5AZA/DAC were responsible for the increased frequency of C > G transversions in leukemic cells following 5AZA/DAC treatment, we used an in vitro Genotoxic Mutational Signature Identified After Clonal Expansion In vitro (GEMINI) assay. This assay is outlined in Fig. 5A, and utilizes single cell sorting following treatment with drug or control. Sequencing of single cell cloned populations allows the detection of mutations that are masked in a bulk population of cells.

Figure 5B summarizes the results of a GEMINI assay using a murine T-ALL cell line (7298) [16] exposed to three concentrations of 5AZA (0.3 uM, 1 uM, and 3 uM). Five individual clones for each treatment group were sequenced and compared to the initial expanded, untreated parental clone using DRAGEN (Supplementary Table S4; Fig. 5B). All three 5AZA concentrations showed increased total mutations and increased C > G transversions compared to the PBS control; increases in C > T and C > A mutations were more variable. Interestingly, we noted a pattern of decreased C > G and total SNV with increasing concentrations of 5AZA. The SBS variants from each group were then pooled and analyzed by SigProfiler as reported above. SBS profiles and decomposition plots of 5AZA treated cells again revealed a similar profile to that observed in T-ALL arising in 5AZA/DAC treated mice in vivo (Fig. 5C, D, Supplementary Fig. S5), with decomposition plots involving SBS39 and SBS98 showing a cosine similarity of 0.88. The full data set can be found in Supplementary Table S4.

**Fig. 6:**
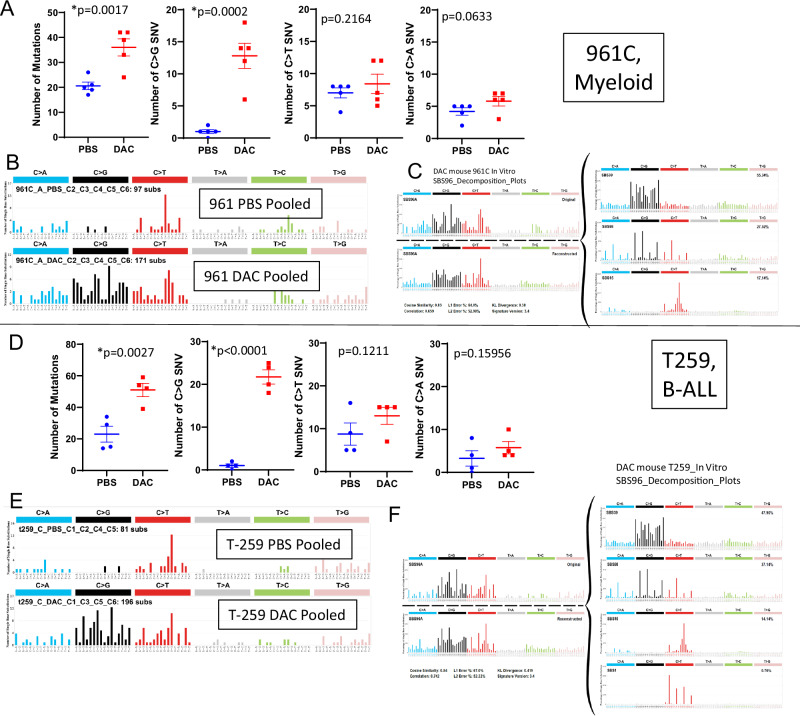
In vitro DAC treatment reveals increased C > G transversions and mutation signature similar to 5AZA treatment. **A** DAC treatment of myeloid cell line (961C) leads to increased total mutations and C > G transversions, **B** SBS signature similar to that seen with 5AZA, and **C** decomposition plot similar to 5AZA. **D** DAC treatment of B-lymphoid cell line (T259) leads to increased total mutations and C > G transversions, **E** SBS signature similar to that seen with 5AZA, and **F** decomposition plot similar to 5AZA.

Given the structural similarity between 5AZA and DAC, we utilized the GEMINI assay to assess the mutagenic potential of DAC in murine cell lines of myeloid (961C) or B-cell (T259) origin (Supplementary Table S5). The 961C myeloid cell line demonstrated increased total mutations and C > G transversions (Fig. 6A) and a SBS mutation signature similar to that seen with 5AZA (Fig. 6B, Supplementary Fig. S6). Similar to findings with 5AZA, the decomposition suggested contributions principally from SBS39 and SBS98 (Fig. 6C). Findings with the T259 B lymphoid cell line treated with DAC also showed increased C > G transversions and similar mutation signature as that seen with 5 AZA (Fig. 6D–F). Taken together, these results demonstrate that the mutation signatures produced by both DAC and 5AZA are quite similar, and the mutational process is not restricted to T-cells, but can be seen in myeloid and B cells as well.

### Increased C > G transversions in human cell lines treated with 5AZA or DAC in vitro

**Fig. 7:**
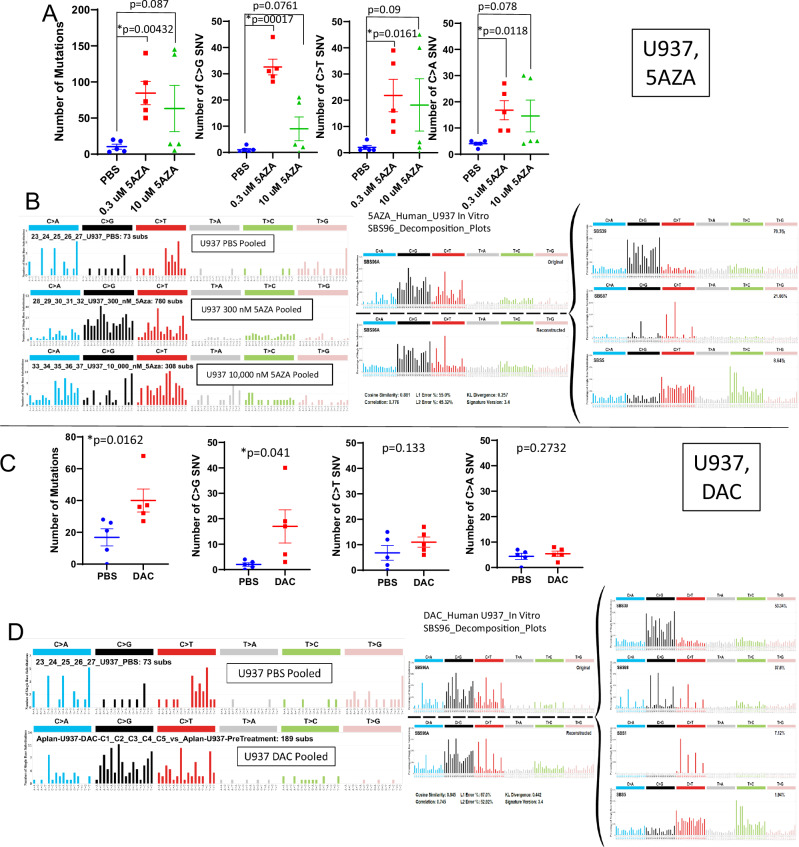
5AZA or DAC treatment generates C > G transversions in human AML cells in vitro. **A** WES reveals an increase of total mutations, C > G, and C > T in 5AZA treated U937 cells with an inverse dose-response relationship. **B** SBS profile of 5AZA treated U937 cells. **C** WES reveals an increase of total mutations and C > G transversions in DAC treated U937 cells. **D** SBS profile of DAC treated U937 cells.

We used the GEMINI assay (Fig. 5A) to assess mutagenicity of 5AZA in U937, a human monocytic leukemia cell line (Supplementary Table S6). Similar to the murine cell lines, we noted an increased number of C > G transversions, again with an inverse dose-response relationship (Fig. 7A). The mutation signature at the lower (300 nM) concentration showed that the increase in C > G transversions was not in a specific trinucleotide context, and the decomposition plot identified similarity (cosine similarity of 0.88) to SBS39 and SBS87 (Fig. 7B, Supplementary Fig. S7).

Treatment of U937 cells with DAC revealed similar findings; an increase in total SNV, due primarily to an increase in C > G transversions (Fig. 7C, Supplementary Table S7). Mutation signature analysis again showed a predominance of C > G transversions in no specific trinucleotide context, and cosine similarity of 0.845 to SBS39 and SBS98 (Fig. 7D, Supplementary Fig. S8).

### Molnupiravir is not mutagenic in human AML cells in vitro

**Fig. 8:**
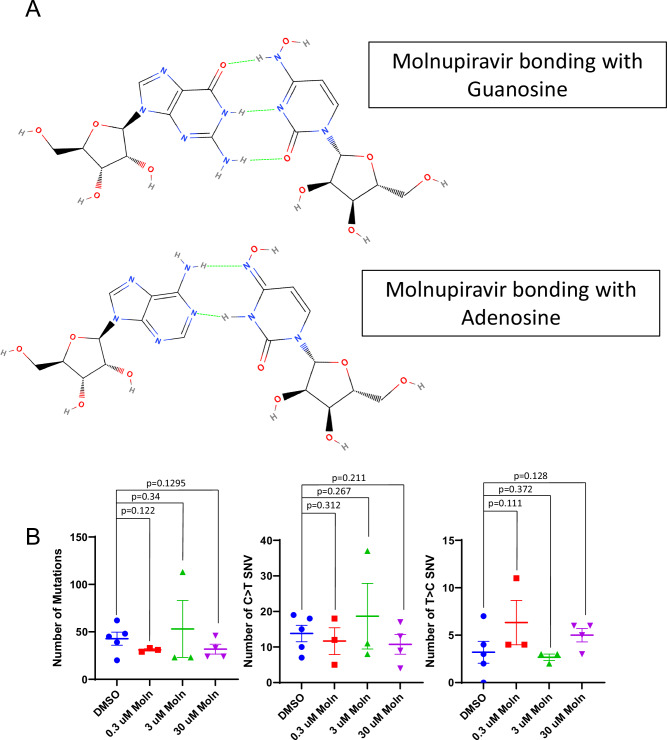
Molnupiravir is not mutagenic in human AML cells in vitro. **A** Hypothetical mispairing of molnupiravir with guanosine and adenosine, allowing for C > T and T > C mutations in RNA. **B** Total, C > T and T > C mutations in molnupiravir-treated U937 cells.

Given the above findings for 5AZA and DAC, as well as previously published findings for ATC [19], we asked whether molnupiravir, a cytidine analog used as an investigational agent for the treatment of Covid19 infections [29], could be mutagenic in mammalian cells. Molnupiravir is proposed to cause lethal mutagenesis in RNA viruses due to C > T and T > C mutations induced by hydrogen bonding to both adenosine and guanosine [30] (Fig. 8A). Although there were suggestions that molnupiravir may be mutagenic in mammalian cells using a single gene (hypoxanthine phosphoribosyltransferase 1 or *HPRT1*) assay [31], we considered that the GEMINI assay (Fig. 5A) would provide mutagenicity information on a genome wide scale, as well as information on the types of mutations. We bracketed an LD50 value of 3 uM based on a similar value reported in CEM cells, a human lymphoid cell line [32]. As shown in Fig. 8B, molnupiravir treatment did not lead to increased mutagenesis using the in vitro GEMINI assay.

## Discussion

In this study, we noted that 5AZA treatment, with or without alvocidib, led to T-ALL in ~20% of mice. These T-ALL were linked to a marked global increase in C > G transversions as compared to AML that arose in the control group. C > G transversions were noted in several genes known to be relevant for T-ALL, including *Stat5b, Ntrk2*, and *Trp53*. In addition, in vitro treatment of both murine and human cell lines with 5AZA using a single cell GEMINI assay led to a clear and dramatic increase in C > G transversions. Although it remains possible that concurrent treatment of some mice with alvocidib may have influenced the mutational process, the fact that the increased C > G signature from murine cells treated solely with 5AZA in vitro was indistinguishable from the dual 5AZA/ALVO treatment in vivo strongly suggests that alvocidib did not play a role.

It is unclear why we did not see a therapeutic benefit of 5AZA in the cohorts of NHD13 mice that we treated. It may be that a benefit was negated by increased toxicity (anemia, thrombocytopenia, neutropenia in additional to T-ALL) or disease progression. We have shown previously that NHD13 mice acquire somatic mutations in tyrosine kinase and Ras signaling pathways [33]; it is possible that acquisition of specific mutations decreases the efficacy of 5AZA in this model of MDS. Moreover, over 80% of 5AZA is incorporated into RNA rather than DNA, and the true mechanism underlying the efficacy of 5AZA in MDS remains unclear [34**–**36].

The demonstration that 5AZA can be mutagenic and oncogenic is not completely surprising. We recently published a paper which demonstrated that ATC, an investigational thiol derivative of DAC, is both mutagenic and oncogenic in mice [19], and a prior publication showed that 5AZA could be mutagenic in murine colon cells [37]. Moreover, an earlier study demonstrated that 5AZA treatment at 2.0 mg/kg ×50 weeks was linked to lymphoid (not immunologically evaluated), lung, mammary, and skin tumors [38]. However, the current experiments demonstrate a direct link between an FDA approved drug (5AZA), a mutation signature, and cancer.

We were able to extend the findings of increased C > G transversions following 5AZA treatment to DAC, a deoxy derivative of 5AZA, as treatment of the human U937 cell line with DAC in vitro led to a clear increase in C > G transversions. Moreover, in mice, we showed that the increase in C > G transversions was not limited to T cell precursors, but was also see in association with murine AML in vivo (albeit a single case), as well as myeloid and B-lymphocyte cell lines in vitro. Taken together, these studies clearly demonstrate the mutagenic potential of these aza-nucleosides. Not all cytidine analogs display this increase in C > G mutagenesis; for instance, analysis of molnupiravir using an identical GEMINI assay failed to show any increase in mutation frequency over background. However, it should be noted that molnupiravir does not contain an aza motif in the cytosine ring, and therefore may not be subject to the same mechanism of mutagenesis observed with aza-nucleosides.

Although the increase in C > G transversions was the most pronounced and consistent mutagenic finding following 5AZA or DAC treatment, an increase in C > T and C > A mutations following 5AZA or DAC was also seen in several experiments. The reason that this was not a consistent finding is unclear, but may be related to the size of the data set, as the data sets with the highest total number of SNV (T-ALL in vivo and U937 5AZA in vitro) both showed a consistent increase in C > T and C > A mutations. We analyzed the mutation signatures produced by 5AZA and DAC, both in vitro and in vivo. SigProfiler and Extractor software consistently identified similarity to SBS39 and SBS98, with cosine similarity of 0.80-0.88. SBS39 has no known etiology, and has previously been identified in a small number of patients with medulloblastoma, prostate, and breast cancer [39], whereas SBS98 (previously designated SBS105) has no known etiology, and has been identified in a small number of patients with bladder, breast, and kidney cancer [28, 40]. As noted, the SBS98 signature strongly resembles a recently reported signature (SBS ATC) which is caused by treatment with ATC, an investigational agent with a structure identical to DAC except a thiol substitution in the deoxyribose ring [19].

The mechanism leading to mutagenesis caused by 5AZA and DAC is unclear. A previous report has suggested a DNMT1-dependent mechanism, in which 5AZA is metabolized to 5-aza-2’-deoxycytidine triphosphate via the ribonucleotide reductase pathway, and subsequently incorporated into a newly synthesized strand of DNA, resulting in a hemi-methylated strand of DNA (the 5-aza-2’-deoxycytidine incorporated into DNA is not methylated). DNMT1 then covalently binds to the C6 position of the 5-aza-2’-deoxycytidine residue that has been incorporated into the hemimethylated DNA strand [37]. The C5 methylation of 5-aza-2’-deoxycytidine cannot occur as the C5 has been replaced by a nitrogen at position 5 of the 5-aza-2’-deoxycytidine ring, and a protein-DNA adduct is formed. Degradation of this adduct leads to depletion of DNMT1, as well as an opening of the 5-aza-2’-deoxycytidine ring, allowing mispairing of hydrogen bonds now pairing a (ring-open) 5-aza-2’-deoxycytidine to a cytidine; the next round of DNA replication leads to a C > G transversion. Our data is consistent with this proposed mechanism, although our data suggests that while C > G transversions at CpG residues are favored, C > G transversions at non CpG residues are more common, for instance, in our deepest dataset (5AZA treatment in vivo; Fig. 4B) 229/502 (45%) of C > G transversions were in a CpG context, whereas 273/502 (55%) of C > G transversions were in a non-CpG context. This can be explained by 5-aza-2’-deoxycytidine ring-opening at non-CpG residues, through spontaneous hydrolysis of an azacytidine ring [41]. The more modest increase in C > T and C > A mutations seen following 5AZA or DAC treatment could be explained by more promiscuous mispairing of A or T residues to a ring-opened 5-aza-2’-deoxycytidine residue or repair of an abasic site following removal of a ring-opened 5-aza-2’-deoxycytidine.

Taken together, these results show that 5AZA is leukemogenic in mice, likely through a mutagenic effect centered on cytidine residues. The mutagenic effect of 5AZA and DAC can be clearly recapitulated in vitro, in both human and murine cells, using a single cell GEMINI assay. Although lymphoid leukemia is uncommon in MDS or AML patients treated with 5AZA or DAC [42], increased C > G mutations have been observed in MDS and AML patients treated with DAC [19, 43]. Further study and continued monitoring of patients treated with 5AZA or DAC would seem to be warranted.

## Supplementary information

Supp Figures S1-S8

Supp Tables S1-S7

## Data Availability

The data generated in this study are available within the article and its supplementary data files. Next generation sequence data for mice and cell lines (mouse and human) are available at the Single Read Archive (SRA), project number PRJNA1257440.
