# Supplementary material for: 5-Azacytidine and decitabine induce C > G transversions in both murine and human cells
Source: Leukemia. 2025 Jul 18;39(9):2112–24. doi: 10.1038/s41375-025-02670-y (PMC12380594; doi:10.1038/s41375-025-02670-y)
Supplement: Supplementary file 1 — Supp Figures S1-S8 [file 41375_2025_2670_MOESM1_ESM.pptx]

## Slide 1
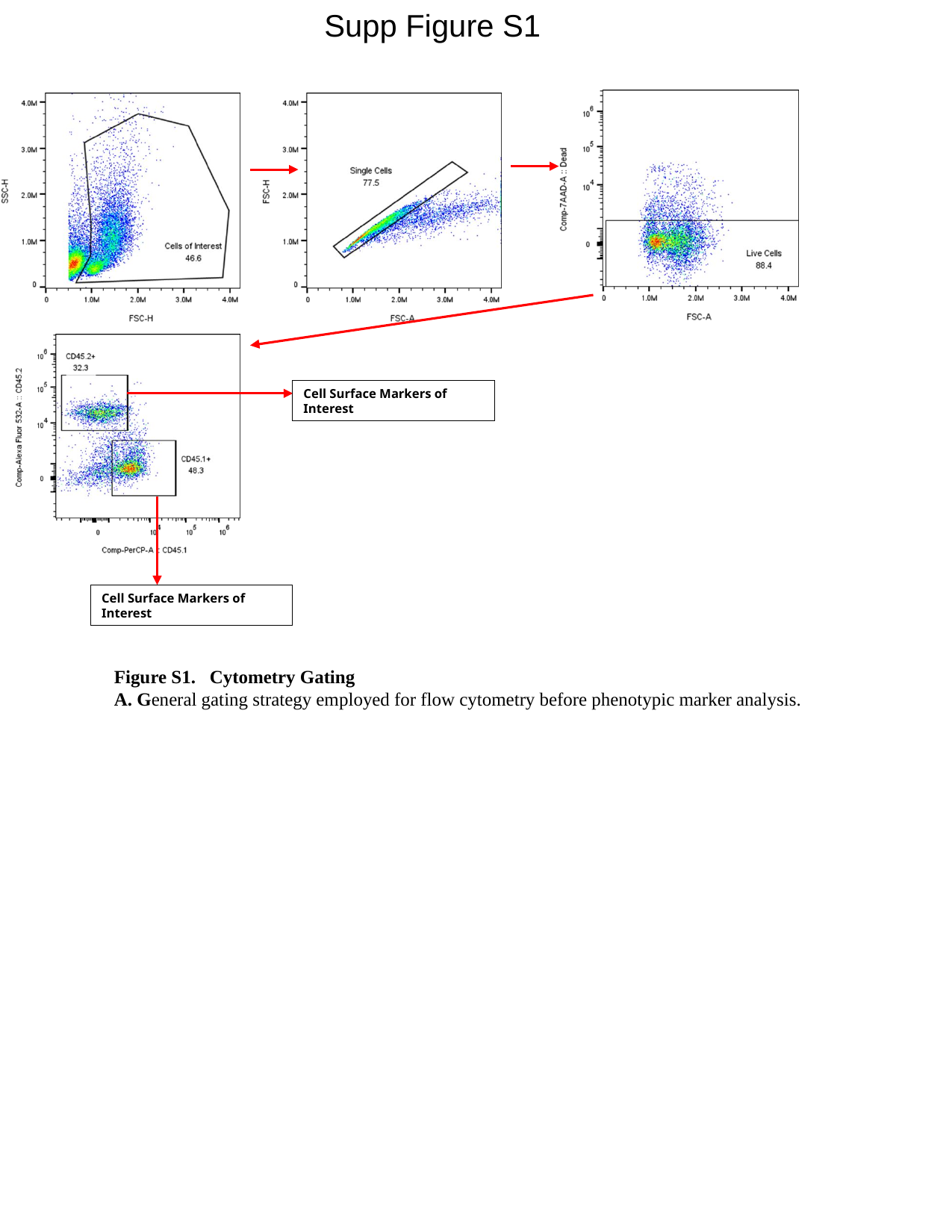

Supp Figure S1
Cell Surface Markers of Interest
Cell Surface Markers of Interest
Figure S1. Cytometry Gating
A. General gating strategy employed for flow cytometry before phenotypic marker analysis.

## Slide 2
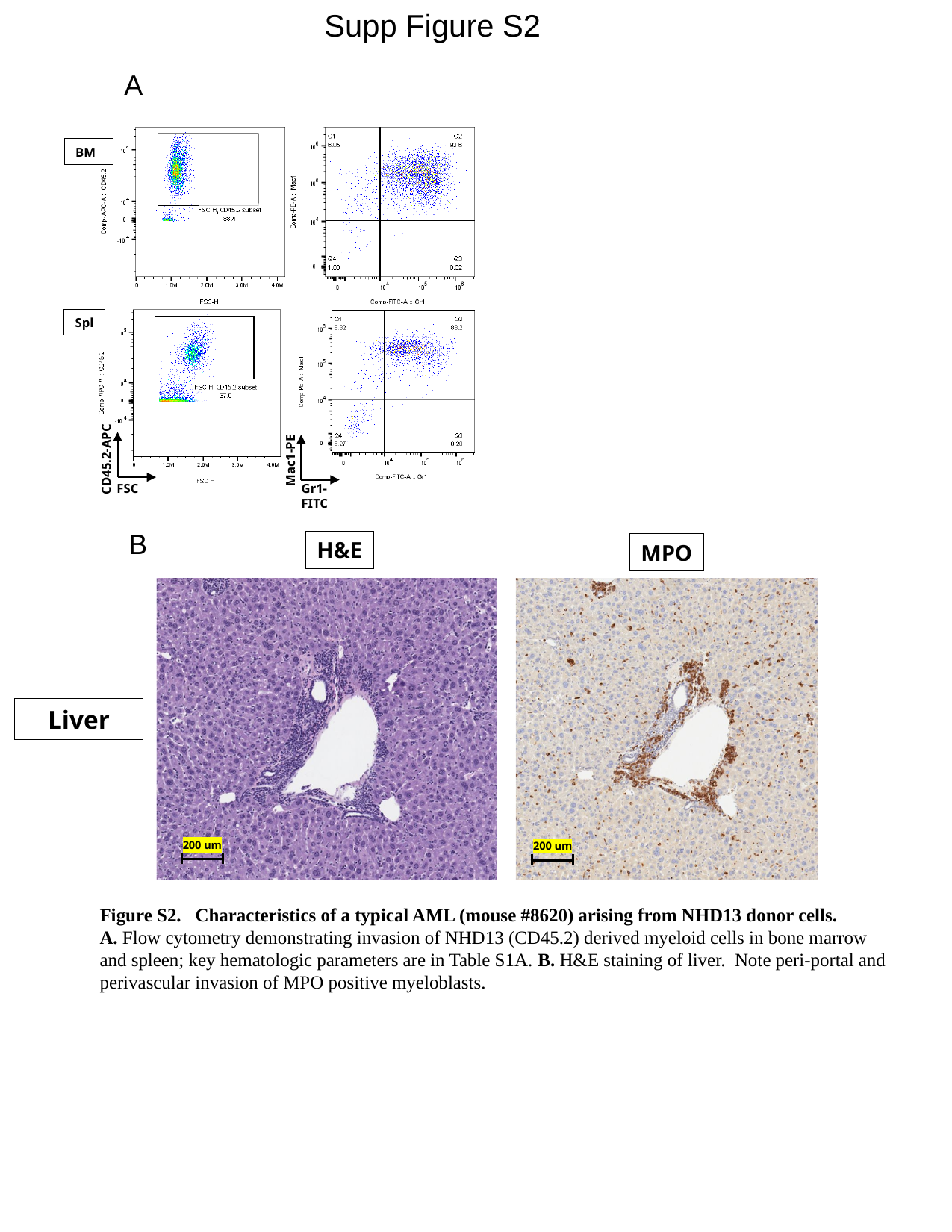

Supp Figure S2
A
BM
Spl
CD45.2-APC
Mac1-PE
Gr1-FITC
FSC
B
H&E
MPO
Liver
200 um
200 um
Figure S2. Characteristics of a typical AML (mouse #8620) arising from NHD13 donor cells.
A. Flow cytometry demonstrating invasion of NHD13 (CD45.2) derived myeloid cells in bone marrow and spleen; key hematologic parameters are in Table S1A. B. H&E staining of liver. Note peri-portal and perivascular invasion of MPO positive myeloblasts.

## Slide 3
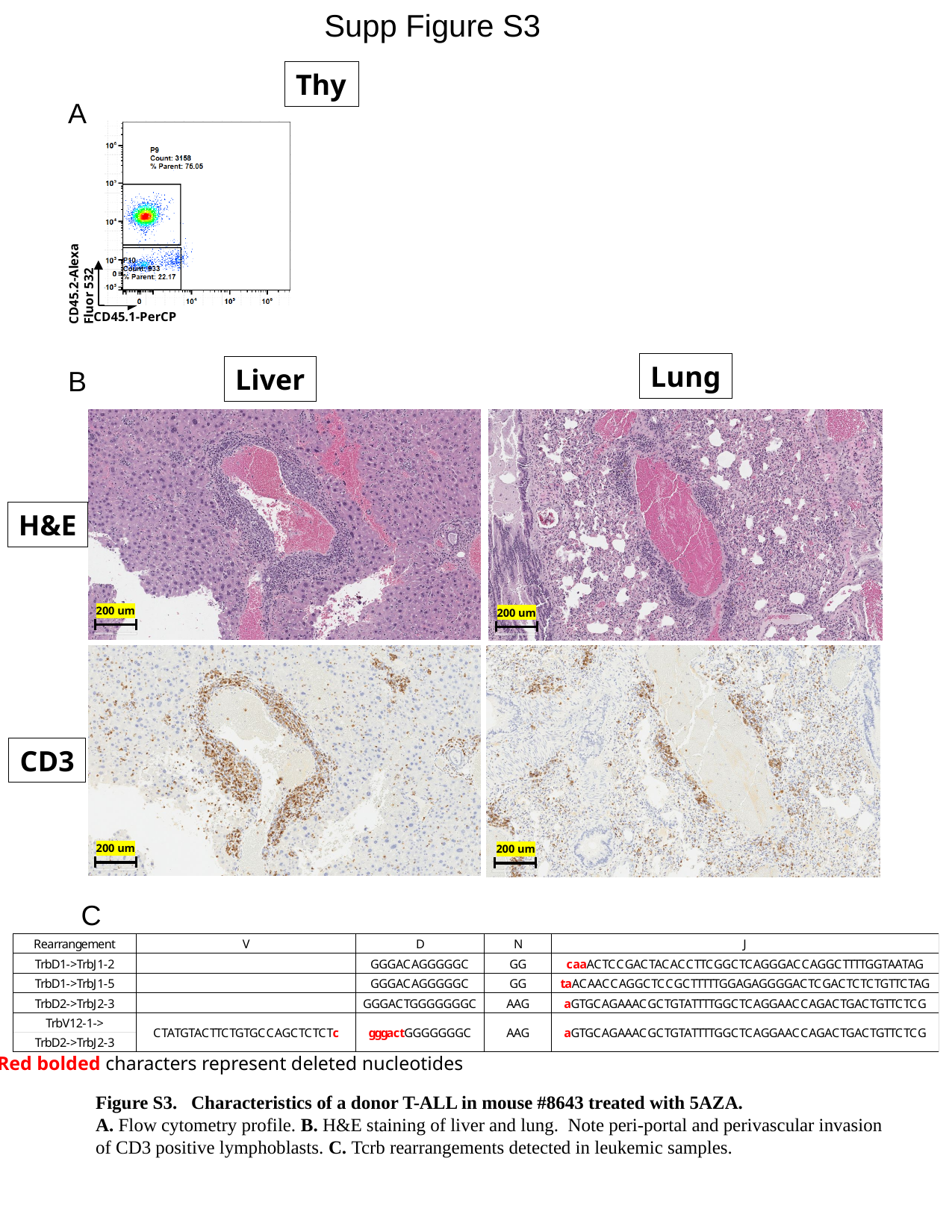

Supp Figure S3
Thy
A
CD45.2-Alexa
Fluor 532
CD45.1-PerCP
Lung
Liver
B
H&E
200 um
200 um
CD3
200 um
200 um
C
* Red bolded characters represent deleted nucleotides
Figure S3. Characteristics of a donor T-ALL in mouse #8643 treated with 5AZA.
A. Flow cytometry profile. B. H&E staining of liver and lung. Note peri-portal and perivascular invasion of CD3 positive lymphoblasts. C. Tcrb rearrangements detected in leukemic samples.

## Slide 4
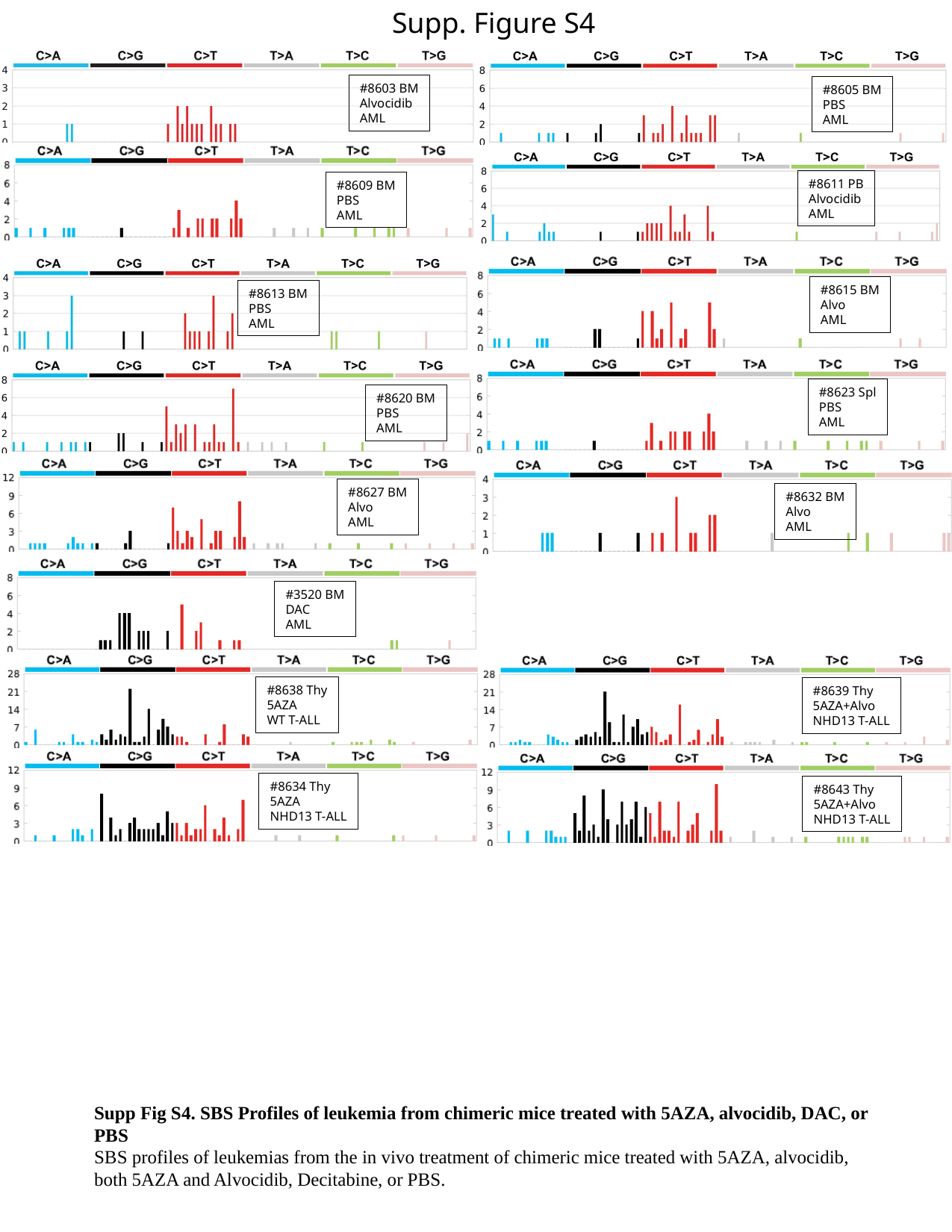

Supp. Figure S4
#8603 BM
Alvocidib
AML
#8605 BM
PBS
AML
#8611 PB
Alvocidib
AML
#8609 BM
PBS
AML
#8615 BM
Alvo
AML
#8613 BM
PBS
AML
#8623 Spl
PBS
AML
#8620 BM
PBS
AML
#8627 BM
Alvo
AML
#8632 BM
Alvo
AML
#3520 BM
DAC
AML
#8638 Thy
5AZA
WT T-ALL
#8639 Thy
5AZA+Alvo
NHD13 T-ALL
#8634 Thy
5AZA
NHD13 T-ALL
#8643 Thy
5AZA+Alvo
NHD13 T-ALL
Supp Fig S4. SBS Profiles of leukemia from chimeric mice treated with 5AZA, alvocidib, DAC, or PBS
SBS profiles of leukemias from the in vivo treatment of chimeric mice treated with 5AZA, alvocidib, both 5AZA and Alvocidib, Decitabine, or PBS.

## Slide 5
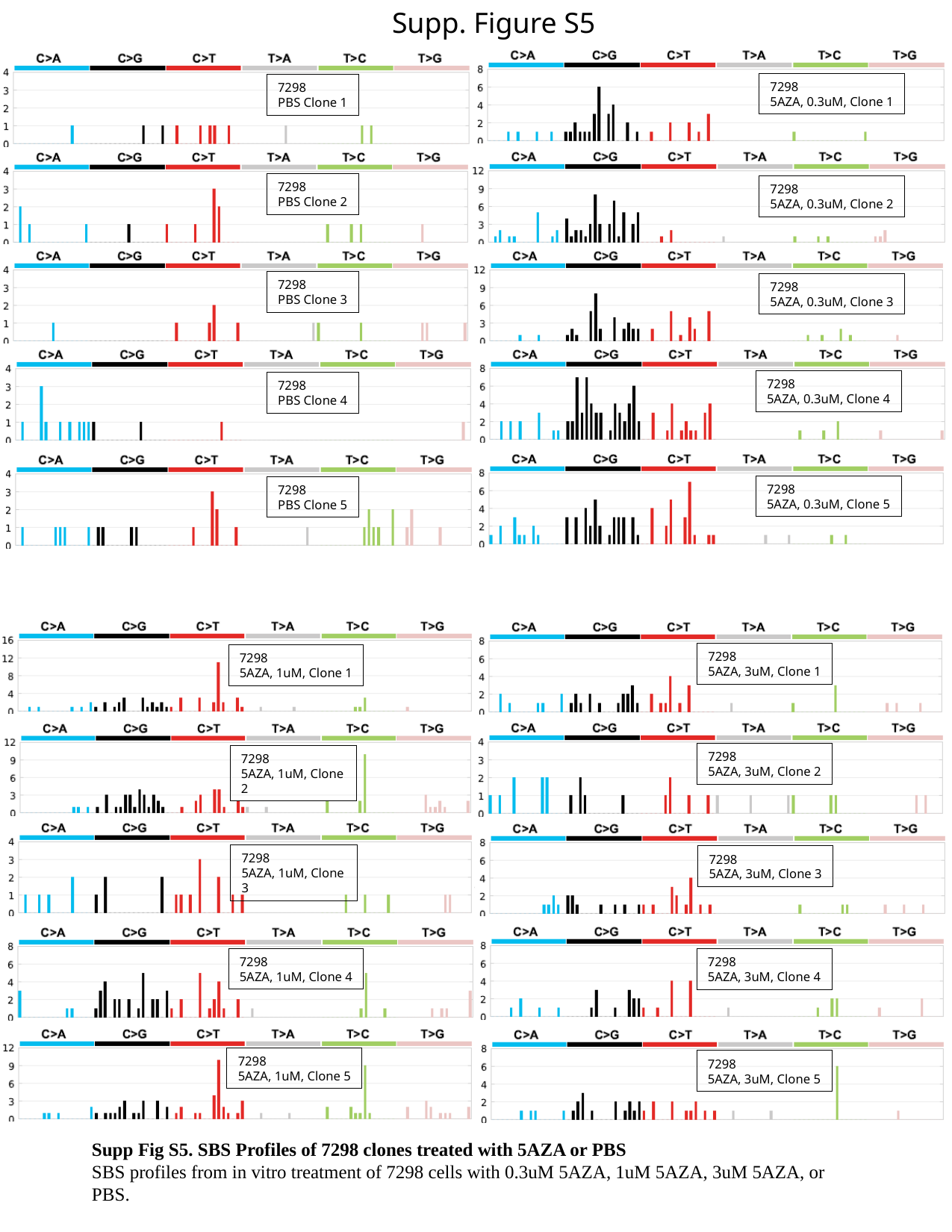

Supp. Figure S5
7298
5AZA, 0.3uM, Clone 1
7298
PBS Clone 1
7298
PBS Clone 2
7298
5AZA, 0.3uM, Clone 2
7298
PBS Clone 3
7298
5AZA, 0.3uM, Clone 3
7298
5AZA, 0.3uM, Clone 4
7298
PBS Clone 4
7298
5AZA, 0.3uM, Clone 5
7298
PBS Clone 5
7298
5AZA, 3uM, Clone 1
7298
5AZA, 1uM, Clone 1
7298
5AZA, 3uM, Clone 2
7298
5AZA, 1uM, Clone 2
7298
5AZA, 1uM, Clone 3
7298
5AZA, 3uM, Clone 3
7298
5AZA, 1uM, Clone 4
7298
5AZA, 3uM, Clone 4
7298
5AZA, 1uM, Clone 5
7298
5AZA, 3uM, Clone 5
Supp Fig S5. SBS Profiles of 7298 clones treated with 5AZA or PBS
SBS profiles from in vitro treatment of 7298 cells with 0.3uM 5AZA, 1uM 5AZA, 3uM 5AZA, or PBS.

## Slide 6
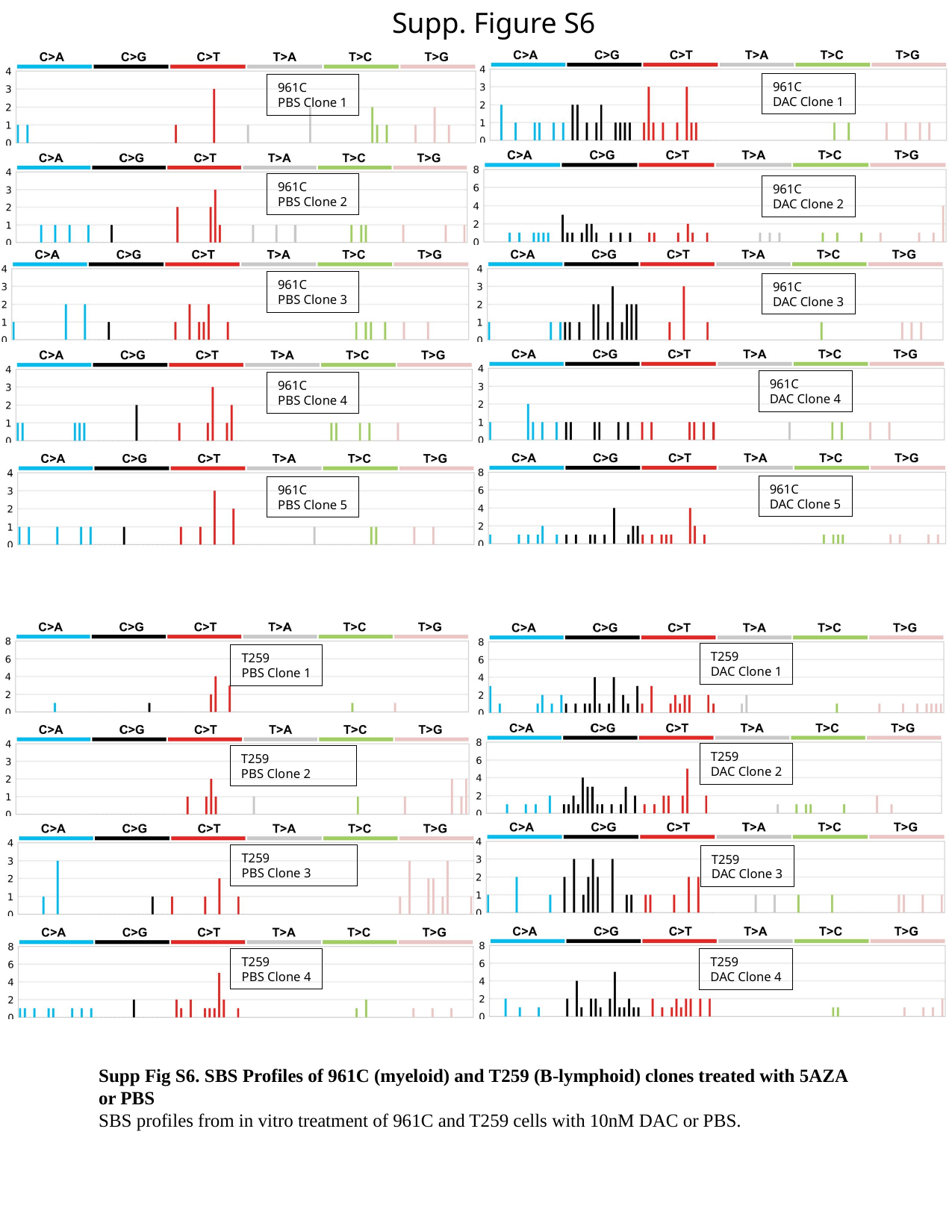

Supp. Figure S6
961C
DAC Clone 1
961C
PBS Clone 1
961C
PBS Clone 2
961C
DAC Clone 2
961C
PBS Clone 3
961C
DAC Clone 3
961C
DAC Clone 4
961C
PBS Clone 4
961C
DAC Clone 5
961C
PBS Clone 5
T259
DAC Clone 1
T259
PBS Clone 1
T259
DAC Clone 2
T259
PBS Clone 2
T259
PBS Clone 3
T259
DAC Clone 3
T259
PBS Clone 4
T259
DAC Clone 4
Supp Fig S6. SBS Profiles of 961C (myeloid) and T259 (B-lymphoid) clones treated with 5AZA or PBS
SBS profiles from in vitro treatment of 961C and T259 cells with 10nM DAC or PBS.

## Slide 7
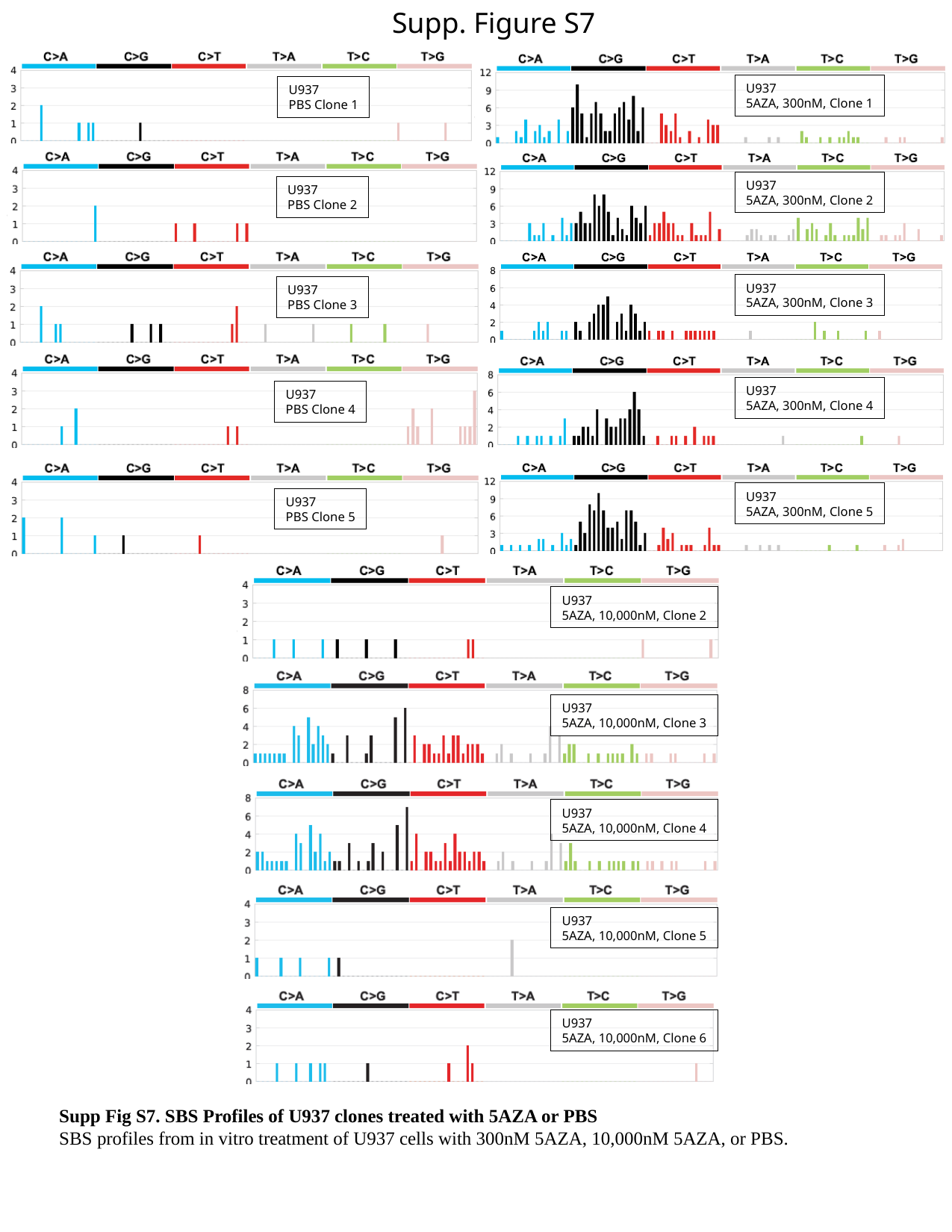

Supp. Figure S7
U937
5AZA, 300nM, Clone 1
U937
PBS Clone 1
U937
5AZA, 300nM, Clone 2
U937
PBS Clone 2
U937
5AZA, 300nM, Clone 3
U937
PBS Clone 3
U937
5AZA, 300nM, Clone 4
U937
PBS Clone 4
U937
5AZA, 300nM, Clone 5
U937
PBS Clone 5
U937
5AZA, 10,000nM, Clone 2
U937
5AZA, 10,000nM, Clone 3
U937
5AZA, 10,000nM, Clone 4
U937
5AZA, 10,000nM, Clone 5
U937
5AZA, 10,000nM, Clone 6
Supp Fig S7. SBS Profiles of U937 clones treated with 5AZA or PBS
SBS profiles from in vitro treatment of U937 cells with 300nM 5AZA, 10,000nM 5AZA, or PBS.

## Slide 8
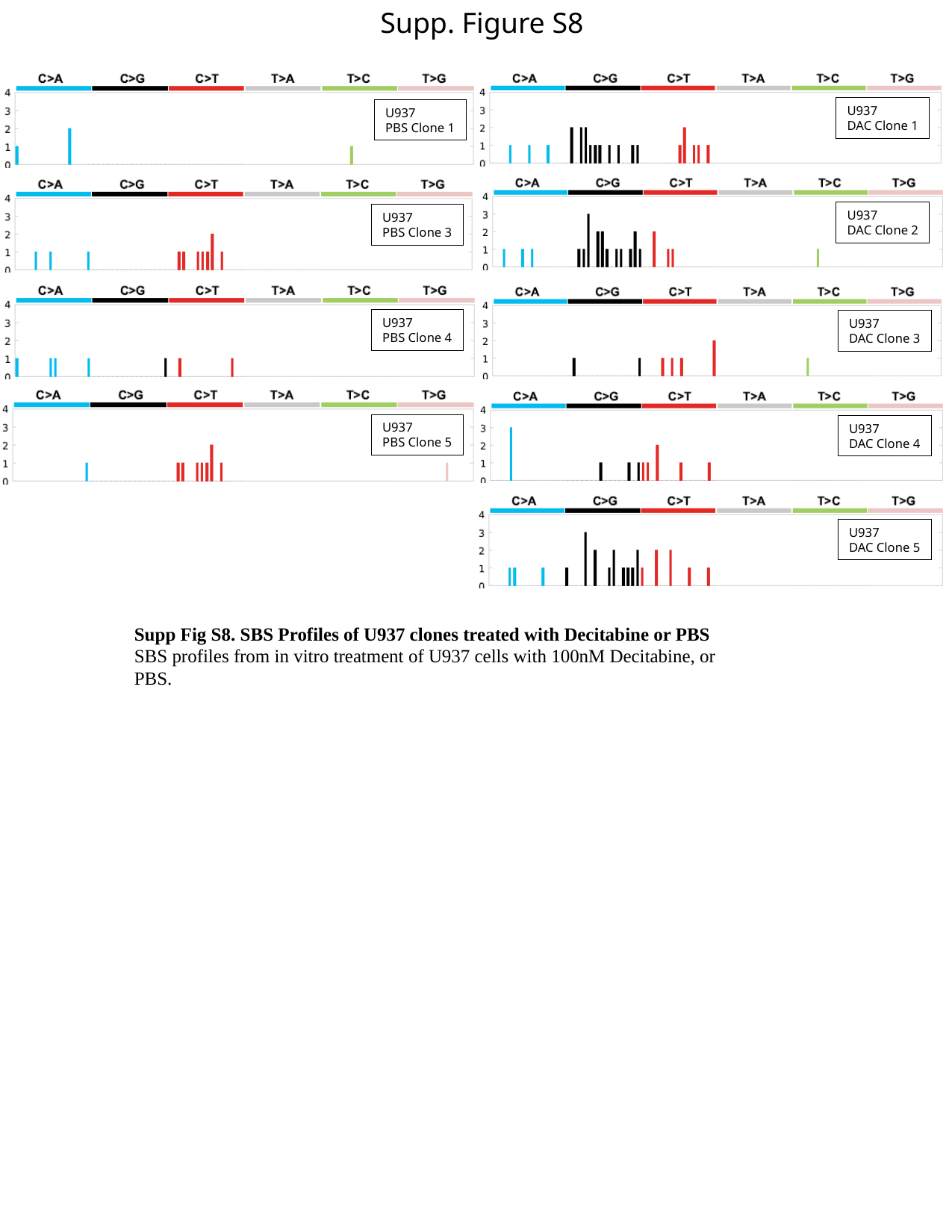

Supp. Figure S8
U937
DAC Clone 1
U937
PBS Clone 1
U937
DAC Clone 2
U937
PBS Clone 3
U937
PBS Clone 4
U937
DAC Clone 3
U937
PBS Clone 5
U937
DAC Clone 4
U937
DAC Clone 5
Supp Fig S8. SBS Profiles of U937 clones treated with Decitabine or PBS
SBS profiles from in vitro treatment of U937 cells with 100nM Decitabine, or PBS.
